# Antioxidant and hepatorenal protective effects of bee pollen fractions against propionic acid‐induced autistic feature in rats

**DOI:** 10.1002/fsn3.1813

**Published:** 2020-08-14

**Authors:** Huda S. Al‐Salem, Hanan M. Al‐Yousef, Abdelkader E. Ashour, Atallah F. Ahmed, Musarat Amina, Iman S. Issa, Ramesa Shafi Bhat

**Affiliations:** ^1^ Pharmaceutical Chemistry Department College of Pharmacy King Saud University Riyadh Saudi Arabia; ^2^ Pharmacognosy Department College of Pharmacy King Saud University Riyadh Saudi Arabia; ^3^ Department of Basic Medical Sciences Kulliyyah of Medicine International Islamic University Malaysia Kuantan Malaysia; ^4^ Department of Pharmacognosy Faculty of Pharmacy Mansoura University Mansoura Egypt; ^5^ Biochemistry Department Science College King Saud University Riyadh Saudi Arabia

**Keywords:** antioxidants, bee pollen, hepatorenal biomarkers, phenolic compounds

## Abstract

In the brain, propionic acid (PA) can cross cell membranes and accumulate within cells, leading to intracellular acidification, which may alter neurotransmitter release (NT), communication between neurons, and behavior. Such elevation in levels of PA constitutes a neurodevelopmental metabolic disorder called propionic acidemia, which could clinically manifest as autism. The purpose of this study was to investigate the protective effects of different fractions of bee pollen (BP) on PA‐induced autism in rats, and to evaluate their effects on the expression of liver and renal biomarkers. Groups of rats received treatments of different fractions of BP at a dose of 250 mg/kg of body weight/day for a period of 1 month. Normal control group I and group II were orally administered with phosphate‐buffered saline and propionic acid, respectively, for 3 days. BP contains various health‐promoting phenolic components. Different fractions of BP administered pre‐ and post‐treatment with PA showed significant reduction in the levels of liver and renal biomarkers (*p* < .05). Also, a significant enhancement in the levels of glutathione S‐transferase (GST), catalase CAT), and ascorbic acid (VIT C) was observed. Supplementation with BP significantly reduced biochemical changes in the liver, kidneys, and brain of rats with PA‐induced toxicity. It exhibited protective effects against oxidative damage and reactive oxygen species produced by PA‐induced adverse reactions in rats. Taken together, our study shows that BP possesses protective effects in PA‐induced liver and kidney damage.

## Graphical abstract

Bee pollen is a mixture of flower pollen, nectar, honey, wax, enzymes, and bee secretions. Foraging honey bees collect pollen from plants and transport it to the beehive, where it is stored and used as food for the colony. The present study demonstrates the protective effect of bee pollen to minimize liver, kidney, and brain insufficiencies associated with propionic acid‐induced autism in rat model. Bee pollen contains high percentage of flavonoid and flavonoid glycoside, suggesting that these compounds might be responsible for the protective effects against PA‐induced hepatorenal and brain injuries as well as antioxidant effects.

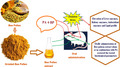

## 1 INTRODUCTION

Bee pollen (BP) has been used for many years as a dietary supplement and as traditional and alternative medicine majorly due to its nutritional value and health benefits (Dubtsova, 2019; Kroyer & Hegedus, 2001). BP is considered as an excellent source of bioactive elements and energy since ancient times. Keeping into account the current demand for natural and healthy food, it is not surprising that BP has been receiving commercial popularity in recent years, making it one of the most widely consumed food supplements. BP is acclaimed the “only perfectly complete food” because it includes all essential amino acids required by a human. Numerous studies have been reported the beneficial effects of the BP as a potential source of natural antioxidants. It is mainly comprised of primary metabolites such as lipids, proteins, sugars, amino acids, vitamins, minerals, and high amounts of polyphenolic components such as flavonoids and phenolic compounds, which possess broad‐spectrum antioxidant and antimicrobial activities (Aabed, Bhat, et al., 2019; Aabed, Shafi Bhat, et al., 2019; Al‐Salem, Bhat, Al‐Ayadhi, & El‐Ansary, 2016; El‐Ansary, Al‐Salem, Asma, & Al‐Dbass, 2017; Kocot, Kiełczykowska, Luchowska‐Kocot, Kurzepa, & Musik, 2018; Komosinska‐Vassev, Olczyk, Kazmierczak, Mencner, & Olczyk, 2015).

Many studies have been reported that BP is effective in treating physical and mental overtiredness, asthenia, and apathy. The administered BP alongside antidepressants enables the lowering doses of drug and improves condition in a short period of time. Also, it improves the blood supply to nervous tissue, boosts mental capacity, and strengthens the nervous system weakened by stress or overworking. This is owing to the nutritional and tonic properties of BP (Denisow & Denisow‐Pietrzyk, 2016; Wójcicki, 1987; Wójcicki, Hinek, & Samochowiec, 1985). It has been found that the long‐term use of BP, even in small doses, improves physical and mental activities (Wójcicki et al., 1985).

The BP extract has a strong antibiotic activity for Gram‐positive bacteria, such as *Staphylococcus aureus*, Gram‐negative bacteria, such as *Escherichia coli*, *Klebsiella pneumoniae*, and *Pseudomonas aeurgionsa*, and fungi such as *Candida albicans*. Also, BP is effectively removing the pain induced by inflammations (Droździk, 1993; Shoskes, 2002; Wu & Lou, 2007; Yasumoto et al., 1995). This is owing to the presence of flavonoids and phenolic acids (Baltrušayt, Venskmonis, & Čeksteryte, 2007; Erkmen & Özcan, 2008). Interestingly, BP possesses an antiallergic activity. It protects mast cells from degranulation due to release of histamine, which is an indicator of allergic reactions. The releasing of histamine from mast cells induced by the serum‐containing anti‐IgE antibodies was inhibited by pollen by 62% (Ishikawa et al., 2008). However, one case study has reported the potential allergic reactions when ingested BP by patients has pollen allergy (Jagdis & Sussman, 2012).

Propionic acid (PA) is found in the diet as a short‐chain fatty acid that is present naturally in milk and milk products such as cheese and yogurt (Lee et al., 2010). It is also derived from colonic bacterial fermentation of oligosaccharides, polysaccharides, proteins, peptides, long‐chain fatty acids, and glycoproteins that contribute mainly to the overall propionate accumulation in children, which is referred to as propionate metabolism disorder (Es, Khaneghah, Hashemi, & Koubaa, 2017). PA is well known for its fungicidal and bactericidal properties, and therefore, it is commonly used as a food preservative (Mani‐Lopez, Garcia, & Lopez‐Malo, 2010). However, in the pharmaceutical industry, PA is often used for the treatment of wound infections, as a component in anti‐inflammatory formulations such as fluticasone inhalers, as a decongestant and antihistamine, as a component of conjunctivitis, and in anti‐arthritic drugs (Britto, 2001). In the gut, microbes and their metabolites such as PA can gain access to the central nervous system through the liver across the blood–brain barrier. Most of the PA loaded into the colon is absorbed, and drained into the portal vein. It is mainly metabolized in the liver and transported in the blood. PA is neurotoxic and gains access to the brain through the liver (Collins, Surette, & Bercik, 2012; Cryan & Dinan, 2012). A recent study found that in the gut bacteria, toxins play a major role in bowel complications in autistic patients. The use of BP showed ameliorative effects on oxidative stress markers such as glutathione (GSH) and ascorbic acid (vitamin C) of PA‐induced changes in hamsters' brain biomarkers. Also, BP is effective to revive normal gut function in autistic patients (Aabed, Shafi Bhat, et al., 2019).

In the brain, PA can cross cell membranes and accumulate within cells, leading to intracellular acidification, which may alter neurotransmitter release (NT), communication between neurons, and behavior (Al‐Salem et al., 2016; El‐Ansary, Bacha, & Kotb, 2012). Such elevation in levels of PA constitutes a neurodevelopmental metabolic disorder called propionic acidemia, which could clinically manifest as autism (Al‐Owain et al., 2013).

Neuroinflammation plays a pivotal role in the etiology and pathogenesis of autism owing to the distribution of cytokine levels in autistic patient brains. A recent study has been indicated that the BP has a protective role in ameliorating neuroinflammation through regulation of cytokine levels, in a rat model of autism (Aabed, Bhat, et al., 2019). Recent study found that BP was effective in ameliorating the neurotoxic signs and impaired circuit of glutamine–glutamate–GABA of PA‐induced glutamate excitotoxicity, which is manifested by increase in glutamate and the glutamate/glutamine ratio, in addition to decrease in GABA, glutamine, and GABA/glutamate ratio (El‐Ansary et al., 2017). The liver is an important organ involved in various metabolic processes. An elevation in the levels of serum marker enzymes because of any oxidative stress is considered one of the most sensitive indices related to hepatic damage (Kocot et al., 2018; Marrocco, Altieri, & Peluso, 2017).

Several animal models have been used to determine the etiology and pathogenesis as well as healing potential in autism. It was found that the use of rat model is best suited to identify the disease due to the availability of scientific laboratory information in terms of genetic and behavioral considerations of various strains (Al‐Diahan & Bhat, 2015; Al‐Salem et al., 2016; Shultz et al., 2008). In addition, the autistic pathogenesis in rat models have been reported via biomarker associated with neuroinflammation (Al‐Salem et al., 2016; El‐Ansary et al., 2012), oxidative stress (MacFabe et al., 2007; Shultz et al., 2008), neurotransmission dysfunction (Al‐Salem et al., 2016; Narita et al., 2002) alongside with behavioral contribution (Ossenkopp et al., 2012).

Globally, the consumption of natural product such as BP has escalated owing to increase in the capability of counteracting some chronic diseases caused by different oxygen radical species. The oxidative stress is underlying the pathogenesis of serious diseases, such as cancer, neurodegenerative disorders, atherosclerosis, and diabetes and also produced as side effects of the majority of drugs. BP could be used safely and securely to ameliorate the chronic degenerative diseases as mechanisms contributing in pathogenesis of autistic lineaments. Keeping in consideration the abovementioned unique chemical and biological profile of BP has encouraged us to investigate the ability of BP as an antioxidant‐rich food supplement to prevent or minimize PA‐induced liver, kidney, and brain insufficiencies in a rat model.

## 2 MATERIALS AND METHODS

### 2.1 Botanical material

Bee pollen (BP) was purchased from the local market of Wadi Al‐Nahil, one of the largest marketing companies in Riyadh, Saudi Arabia, in June 2016 under the trade name of BP, 100% natural BP first elite. A voucher specimen (BP‐0418) has been deposited in the herbarium of the Pharmacognosy Department, College of Pharmacy, King Saud University, Saudi Arabia.

### 2.2 Solvents

Analytical grade solvents used were 95% ethanol, petroleum ether (40–60°C), dichloromethane (CH_2_Cl_2_), methanol (CH_3_OH), ethyl acetate (EtOAc), and *n*‐butanol, which were distilled prior to use. All the solvents were purchased from Sigma‐Aldrich.

### 2.3 Extraction procedure

Bee pollen (500 g) was extracted with 95% alcohol at room temperature for 48 hr, until it was exhausted. The extracts were combined and filtered. The filtrate was evaporated to dryness under reduced pressure, using rotary vacuum at 45°C to yield a dark yellow gummy extract (91.3 g, yield 18.26% w/w). This extract was dissolved in 30% methanol in distilled water (0.5 L). The hydromethanolic solution was successively fractionated with petroleum ether (3 × 0.5 L), CH_2_Cl_2_ (3 × 0.5 L), EtOAc (5 × 0.5 L), and *n*‐butanol saturated with water (5 × 0.5 L). Each fraction was concentrated under reduced pressure to yield solvent‐free residues: BP petroleum ether (PGP, 7 g); BP dichloromethane (PGC, 9 g); BP ethyl acetate (PGE, 25 g); and BP n‐butanol (PGB 15 g). The remaining aqueous layer was concentrated to yield BP aqueous (PGA, 30.5 g).

The concentrated PGE (25 g) was subjected to vacuum liquid chromatography (VLC) using CH_2_Cl_2_:MeOH gradient, to afford four subfractions: BP‐1 to BP‐4. Subfraction BP‐1 (5.6 g) was chromatographed over silica gel (60–120 mesh), and the elution was carried out in increasing polarity with CH_2_Cl_2_, 2% MeOH in CH_2_Cl_2_, and 5% MeOH in CH_2_Cl_2_ and MeOH. The fractions eluted in 5% MeOH in CH_2_Cl_2_ were combined, concentrated, and the residue on repeated crystallization from methanol gave **1** (12.1 mg, yellow amorphous powder). Subfraction PB‐2 (6.2 g) was treated similarly as PB‐1 to yield **2** (5.6 mg, milky white amorphous powder). Column chromatography of subfraction PB‐3 (4.2 g) over silica gel (60–120 mesh, 200 g × 50 × 5 cm dia.) using CH_2_Cl_2_:MeOH gradients yielded **3** (13.2 mg, white amorphous powder). Subfraction PB‐4 (5.5 g) being highly polar was extracted over Sephadex LH‐20 column (100 g × 50 × 3 cm dia.) using MeOH:H_2_O gradient to afford **4** (16 mg, white amorphous powder).

### 2.4 Physical methods

Melting points were measured on a Buchi melting point B‐545 apparatus (Boston Laboratory Equipment) and not corrected. UV spectrum was recorded on a Shimadzu UV‐265 spectrophotometer, in MeOH, and IR spectrum on a Shimadzu Infrared 400 spectrophotometer, in KBr pellets. ^1^H‐NMR ^13^C‐NMR and 2D‐NMR spectra were determined by a Bruker DRX 700 spectrometer, in CDCl_3_ and DMSO‐d_6_. Chemical shifts were measured in d values (ppm) with tetramethylsilane (TMS) as an internal reference. HRESIMS was measured in a MICROMASS Q‐Tof 2 mass spectrometer, Waters Corporation. Vacuum liquid chromatography (VLC) was performed using silica gel 60 (0.04–0.063 mm; 500 g; Merck). Column chromatography (CC): silica gel (Merck, 60–120 mesh) TLC zones were visualized either by exposure to vanillin sulfuric acid, iodine vapor, or under UV light. All evaporations were achieved in a vacuum on a rotary evaporator.

### 2.5 Animals and monitoring

Experiments were performed on 80 young (21‐ to 24‐day‐old) male Western albino rats (weighing 70–80 g each), obtained from the animal house of Pharmacy College, King Saud University, Riyadh, Saudi Arabia. All animals were kept in standard cages and maintained under standard laboratory conditions (temperature 22 ± 2°C and humidity with 12‐hr light/12‐hr dark cycle) with free access to pellet food and water ad libitum throughout the study. The protocols used in this study were approved by the Ethics Committee of Experimental Animal Care Society, King Saud University, and all the experiments were performed in accordance with the guidelines of the National Animal Care and Use Committee, Saudi Arabia.

### 2.6 Pre‐ and post‐treatment of PA‐induced toxicity

**SCHEME 1:**
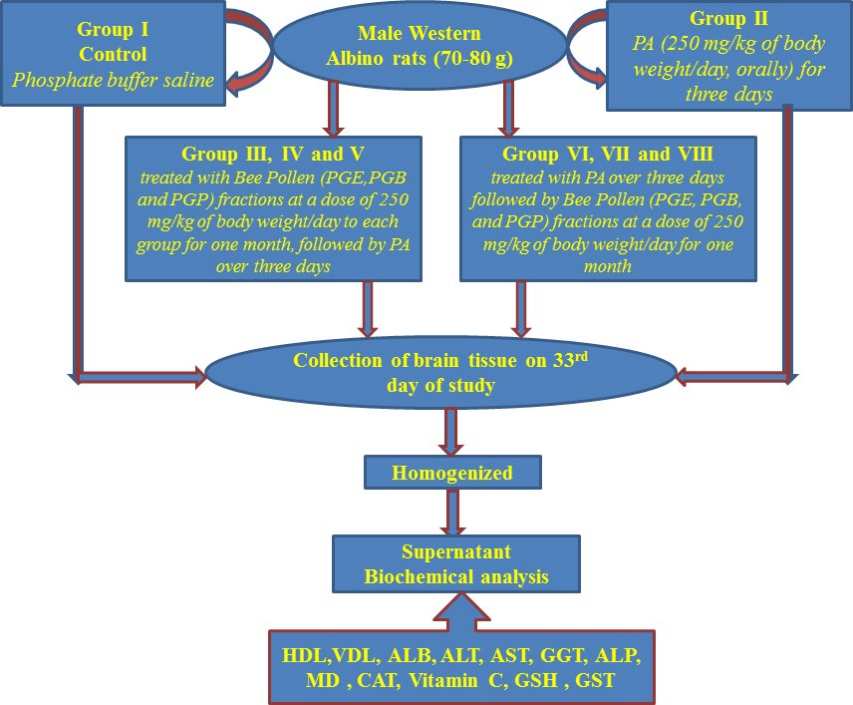
Schematic presentation of the experimental work demonstrating treatment of different studied groups

Male Western albino rats weighing 70–80 g were divided into groups of eight (*n* = 8) for the study. Normal control group I received phosphate‐buffered saline; group II was given PA (250 mg/kg of body weight/day, orally) for 3 days. The prophylactic groups (groups III, IV, and V) received PGE, PGB, and PGP fractions at a dose of 250 mg/kg of body weight/day to each group for 1 month, followed by PA over 3 days. On the other hand, groups VI, VII, and VIII that served as the treatment groups received PA over 3 days followed by PGE, PGB, and PGP fractions at a dose of 250 mg/kg of body weight/day for 1 month. The rats were anesthetized using CO_2_ and then decapitated. Blood samples were collected from retro‐orbital plexus and centrifuged at 1,000 *g* for 15 min at 4°C to obtain serum for analysis. The collected serum was then transferred into prelabeled tubes, stored on ice, and delivered immediately on the same day of collection to Prince Mutaib Chair for Biomarkers of Osteoporosis (PMCO), Biochemistry Department, Biomarkers Research Program (BRP) laboratory, King Saud University, Riyadh, Saudi Arabia, for immediate storage in a −20°C freezer, pending further analysis. Brain tissues were collected and washed with cold saline and then homogenized in 10‐fold distilled water (v/w) using a Teflon Potter Elvehjem Homogenizer. The homogenate was then centrifuged at 4,000 *g* for 20 min. The supernatant obtained was used for various biochemical assays. All solutions were prepared in degassed water purged with nitrogen, prepared fresh, and used immediately (Scheme 1). The dose of BP (250 mg/kg of body weight/day, orally) used was chosen based on previous studies (Aabed, Bhat, et al., 2019; Aabed, Shafi Bhat, et al., 2019). The dose used in this study is considered as diluted (based on the work of separate fractions derived from the total extract), compared with the previous studies where the dose of BP was used as total extract, in addition to the total extract works synergistically, whilst the PA dose used was chosen in this study based on the previous studies (Aabed, Bhat, et al., 2019; Aabed, Shafi Bhat, et al., 2019; Al‐Diahan & Bhat, 2015; Al‐Salem et al., 2016; El‐Ansary et al., 2017).

### 2.7 Serum biochemical analysis

Lipid profile (HDL and VDL cholesterol; total cholesterol triglyceride, TG), calcium (Ca), albumin (ALB), urea, uric acid (UR AC), alanine aminotransferase (ALT), aspartate aminotransferase (AST), γ‐glutamyl transferase (GGT), and alkaline phosphatase (ALP) were measured using routine laboratory analysis (Konelab). This biochemical analyzer was regularly calibrated before the analysis of all serum samples using quality control standards provided by the manufacturers (Thermo Fisher Scientific).

#### 2.7.1 Hepatic and renal function analysis

Serum levels of liver enzymes ALT, AST, GGT, and ALP were measured calorimetrically using the method outlined by Fiala, Fiala, and Dixon (1972), whereas serum levels of kidney Ca were determined by the o‐cresolphthalein method described by Gitelman (1967), using a CE500 Kit (Crescent Diagnostics) (Fiala et al., 1972; Gitelman, 1967). Urease in urea and uricase in UR AC were estimated according to the methods outlined in Munan, Kelly, PetitClerc, and Billon (1978) and Fossati, Prencipe, and Berti (1980), respectively, using Roche Kits (Roche Diagnostics GmbH) (Fossati et al., 1980; Munan et al., 1978).

#### 2.7.2 Albumin level analysis

Analysis of albumin (ALB) in serum was determined by the method given by Doumas (1975) using the CS600 Kit (Crescent Diagnostics) (Doumas, 1975). This method is based on the formation of a violet complex after peptide bonds react with Cu^2+^ in alkaline media (biuret reaction). Potassium sodium tartrate and potassium iodide solution were added as stabilizers. Absorbance was measured at 546 nm, and the amount of albumin was calculated as absorbance of the sample divided by absorbance of control, multiplied by the concentration of control.

#### 2.7.3 Lipid profile analysis

Blood samples were analyzed for measuring lipid profiles including total cholesterol, HDL and VDL cholesterol, and TG, using autoanalyzer, based on enzymatic colorimetric assays reported by Demacker, Hijmans, Vos‐Janssen, van't Laar, & Jansen, 1980, Burstein and Scholnick (1973), and Foster and Dunn (1973), respectively (Burstein & Scholnick, 1973; Demacker et al., 1980; Foster & Dunn, 1973).

Fluorometry was used to determine total cholesterol in cell lysates using the Amplex Red Cholesterol Assay Kit (Invitrogen) according to manufacturer's protocol. Conditioned media or serum was reduced with 50 mM DTT at 37°C for 15 min, precipitated using 150 g/L sulphosalicylic acid, centrifuged, and supernatant was used for detection and analysis. LDL cholesterol and total cholesterol‐to‐HDL ratio were calculated by the following equations: LDL = total cholesterol – HDL − (0.16 × TG) and total cholesterol/HDL ratio = total cholesterol/HDL cholesterol.

### 2.8 Determination of lipid peroxidation

Lipid oxidation was estimated using the thiobarbituric acid reactive substance (TBARS) assay that measures the concentration of the byproducts of lipid peroxidation such as malondialdehyde (MD), based on the method given by Ruiz‐Larrea, Leal, Liza, Lacort, and de Groot (1994). Samples were heated with TBA at low pH; the pink chromogen was detected and measured as absorbance at 532 nm. The concentration of lipid peroxides was estimated in µmoles/ml using MD extinction coefficient.

### 2.9 Antioxidant status analysis

#### 2.9.1 Catalase (CAT) assay

Catalase assay was performed as described by Chance (1954). A total volume of 3 ml of the reaction mixture, containing 1.5 ml of 0.2 M H_2_O_2_ and catalase enzyme, was prepared. The reaction was initialized by adding H_2_O_2,_ and the change in absorbance was measured at 240 nm for 2 min. The values were detected as µmoles of H_2_O_2_ dissociated/min/dl of brain homogenates, represented as U/dl.

#### 2.9.2 Vitamin C (ascorbic acid) assay

Vitamin C assay was performed according to the method given by Jagota and Dani (1982).

#### 2.9.3 Glutathione (GSH) assay

Glutathione was assayed by the method given by Beutler, Duran, and Kelly (1963), using 5,5′‐dithiobis(2‐nitrobenzoic acid) (DTNB) with sulfhydryl compounds to produce a relatively stable yellow color (Beutler et al., 1963).

#### 2.9.4 Glutathione S‐Transferase (GST) activity

Glutathione S‐Transferase activity was assessed according to the method described by Habig, Pabst, and Jakoby (1974), using an assay kit (BioVision) based on the GST‐catalyzed reaction between GSH, GST substrate, and CDNB (1‐chloro‐2,4‐dinitrobenzene) (Habig et al., 1974).

### 2.10 Statistical analysis

All values are calculated as the arithmetic mean ± standard error of the mean (*M* ± *SE*). Statistical analysis was performed by one‐way ANOVA using GraphPad Prism 5.03 (GraphPad Software, Inc) and Microsoft Excel v.2013, followed by Student's test. *p* < .05 was considered significant.

## 3 RESULTS AND DISCUSSION

### 3.1 Identification of isolated compounds

**FIGURE 1:**
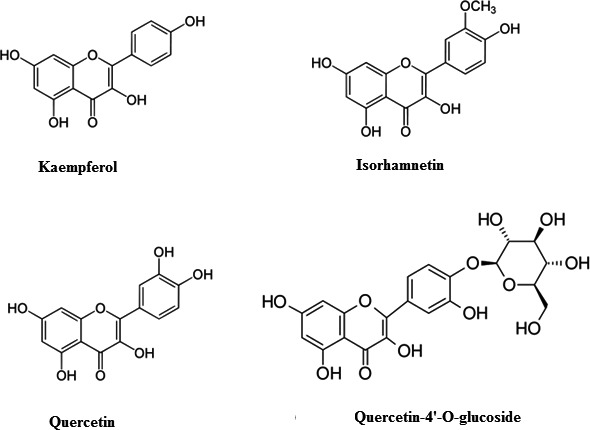
Chemical structures of compounds **1–4**

Compound **1** was isolated as a yellow amorphous powder in 5% methanol in dichloromethane. The HRESIMS analysis of this compound showed a molecular ion peak at *m/z* 286.23 [M+H]^+^ corresponding to the molecular formula C_15_H_10_O_6_, which was identified as kaempferol by comparing its MS, ^1^H‐NMR, and ^13^C‐NMR data with the literature. Compound **2** was isolated as a white amorphous powder in 5% methanol in dichloromethane. The HRESIMS analysis of this compound showed a molecular ion peak at 316.257 [M+H]^+^ (corresponding to C_16_H_12_O_7_) and was identified as isorhamnetin by comparison of its MS, ^1^H‐NMR, and ^13^C‐NMR data with the literature. Compound **3** was isolated as a white amorphous powder in 7% methanol in dichloromethane. The HRESIMS analysis of this compound showed a molecular ion peak at m/z 302.236 [M+H]^+^ (corresponding to C_15_H_10_O_7_, 302.236) and was identified as quercetin by comparison of its MS, ^1^H‐NMR, and ^13^C‐NMR data with the literature. Compound **4** was isolated as a white amorphous powder in 15% methanol in dichloromethane; mp. 310–318°C; IR (KBr) *υ*_max_: 1,660 cm^−1^ for carbonyl functionality, 3,220 cm^−1^ for hydroxyl functionality; and HRESIMS m/z 464.3 [M+H]^+^ (corresponding to C_15_H_10_O_7_, 302.236). It was identified as quercetin‐4′‐O‐glucoside by comparison of its MS, ^1^H‐NMR, and ^13^C‐NMR data with the literature. All compounds gave positive yellow color with FeCl_3_ solution (see Figure 1).

#### 3.1.1 Kaempferol (1)

Yellow amorphous powder; mp. 274–278°C; (IR, KBr) *υ*_max_: 1,565 cm^−1^ for carbonyl functionality; HRESIMS m/z 286.23 [M+H]^+^ (corresponding to C_15_H_10_O_6_, 286.23). ^1^H (DMSO‐d_6_, 700 MHz) δ: 5.41 (1H, H‐6), 7.31 (2H, H‐3′ and H‐5′), 7.48 (1H, H‐8) and 7.82 (2H, H‐2′ and H‐6′). ^13^C NMR (DMSO‐d_6_, 700 MHz) δ: 138.24 (C3), 164.79 (C4), 161.02 (C5), 96.82 (C6), 162.98 (C7), 158.02 (C9), 106.23 (C10), 124.12 (C1′), 128.21 (C2′ and C6′), 118.21 (C3′ and C5′). Based on spectral data and comparison with the literature, compound **1** was identified as kaempferol (Lin, Huang, & Lv, 2016).

#### 3.1.2 Isorhamnetin (2)

White amorphous powder; mp. 302–308°C; (IR, KBr) *υ*_max_: 1,665 cm^−1^ for carbonyl functionality; HRESIMS m/z 316.257 [M+H]^+^ (corresponding to C_16_H_12_O_7_, 316.257). ^1^H (DMSO‐d_6_, 700 MHz) δ: 6.28 (1H, H‐6), 7.48 (1H, H‐2′) 6.75 (1H, H‐5′), and 7.57 (1H, H6′); ^13^C NMR (DMSO‐d_6_, 700 MHz) δ: 156.78 (C2), 134.42 (C3), 176.54 (C4), 162.35 (C5), 98.89 (C6), 164.85 (C7), 93.78 (C8), 158.65 (C9), 105.82 (C10), 124.54 (C1′), 116.78 (C2′), 148.92 (C3′), (C4′)149.84, 116.56 (C5′), and 124.25 (C6′). Compound **2** was identified as isorhamnetin based on spectra and comparison with the literature (Manivannan & Shopna, 2015).

#### 3.1.3 Quercetin (3)

White amorphous powder; mp. 310–318°C; (IR, KBr) *υ*_max_: 1,675 cm^−1^ for carbonyl functionality, 1,478 cm^−1^ for the aromatic group; HRESIMS m/z 302.236 [M+H]^+^ (corresponding to C_15_H_10_O_7_, 302.236). ^1^H(DMSO‐d_6_, 700 MHz) δ: 6.78 (2H, d, H‐6), 6.45 (1H, d, H‐8), 7.18 (1H, d, H‐2′), 6.82 (1H, d H‐5′), 6.89 (1H, d, H‐6′); 13C NMR (700 MHz, DMSO‐d6) δ: 98.75 (C8), 103.12 (C6), 105.3 (C10), 116.28 (C2′ and C5′), 118.78 (C6′), 126.25 (C1′), 131.98 (C3), 145.25 (C3′), 158.68 (C9), 165.32 (C5), 166.84 (C7), 190.54 (C‐4). Compound **3** was identified as quercetin based on spectra and comparison with the literature (Manivannan & Shopna, 2015).

#### 3.1.4 Quercetin‐4′‐O‐glucoside (4)

White amorphous powder; mp. 310–318°C; (IR, KBr) υ_max_: 1,660 cm^−1^ for carbonyl functionality, 3,220 cm^−1^ for hydroxyl functionality; HRESIMS m/z 464.3 [M+H]^+^ (corresponding to C_15_H_10_O_7_, 302.236). ^1^H(DMSO‐d_6_, 700 MHz) δ: 1.305 (2H, dd, 6″H), 3.310–3.698 (4H, H‐2″, H‐3″, H‐4″, H‐5″), 6.21 (1H, d, H‐8′), 6.4 (1H, d H‐6′), 6.88 (1H, d, H‐5′), 7.62 (1H, d, H‐6′) and 7.71 (1H, d, H‐2′); 13C NMR (700 MHz, DMSO‐d6) δ:: 159.2(C2), 134.5(C3), 182.5(C4), 162.9(C5), 102.2(C6), 165.5(C7), 98.5(C8), 161.2(C9), 106.5(C‐10), 124.5(C‐1), 116.5(C‐2), 145.8(C3), 148.2(C4), 122.5(C5), 121.5(C6), 105.2(C1), 76.5(C2), 78.4(C3′), 72.5(C4′), 76.5(C5′), and 64.8(C6′). Based on spectral data and comparison with the literature, compound **4** was identified as quercetin‐4′‐O‐glucoside (Manivannan & Shopna, 2015).

### 3.2 Biochemical analysis

**FIGURE 2:**
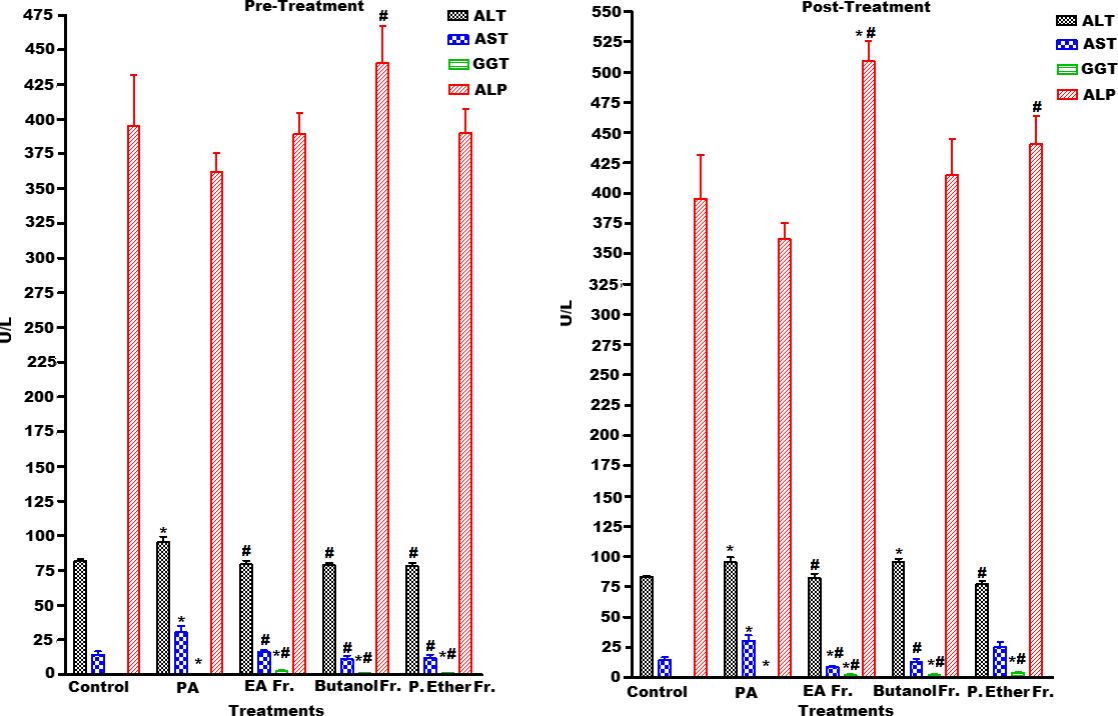
Effect of pre‐ and post‐treatment with PGE, PGB, and PGP on PA‐induced hepatic injury‐related liver serum enzyme markers in rats. All values represent mean ± *SEM*. Statistical analysis was performed using GraphPad Prism 5.03 (GraphPad Software, Inc) and Microsoft Excel v.2013. *p* < .05 was considered significant. *As compared to the control group. ^#^As compared to the PA group, *n* = 8

**FIGURE 3:**
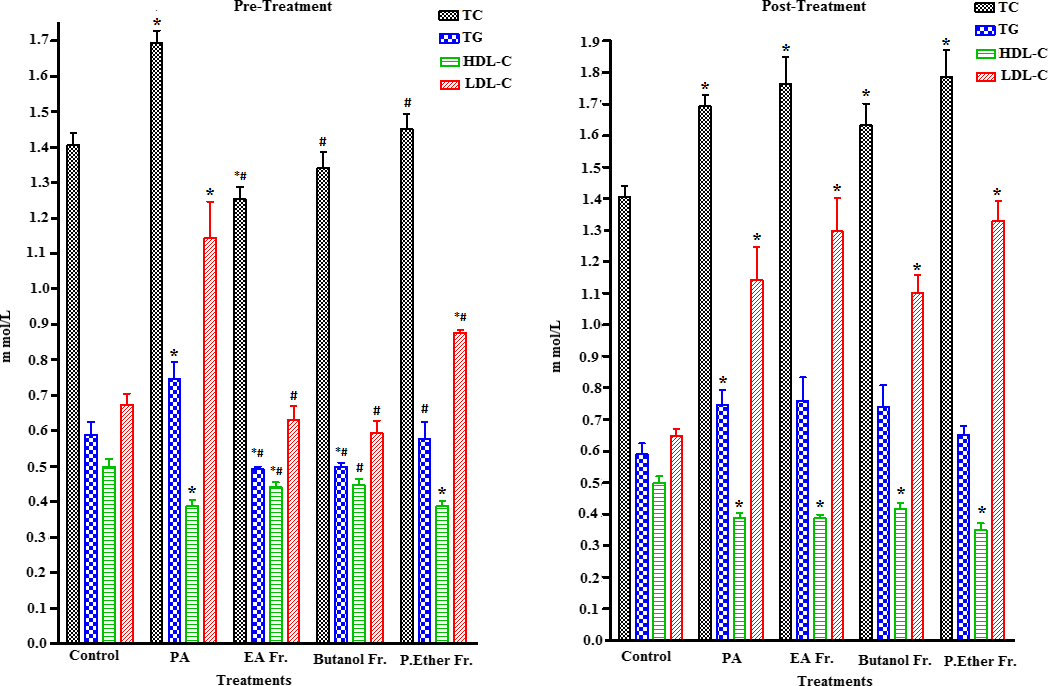
Effect of pre‐ and post‐treatment with PGE, PGB, and PGP on PA‐induced lipid profile changes in rats. All values represent mean ± *SEM*. Statistical analysis was performed using GraphPad Prism 5.03 (GraphPad Software, Inc) and Microsoft Excel v.2013. *p* < .05 was considered significant. *As compared to the control group. ^#^As compared to the PA group, *n* = 8

**FIGURE 4:**
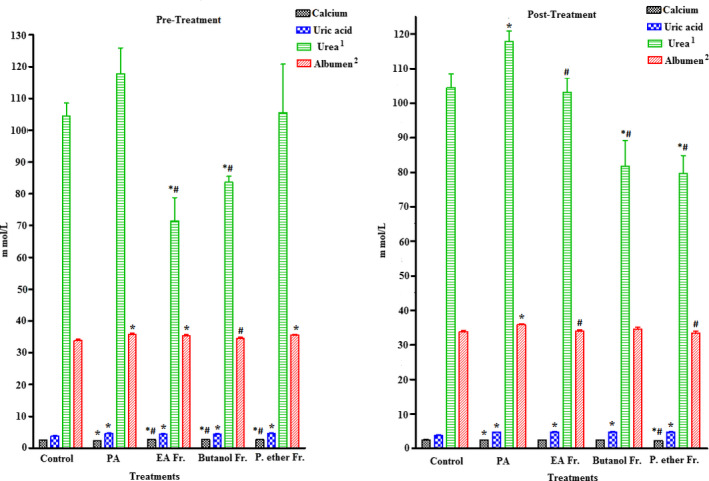
Effect of pre‐ and post‐treatment with PGE, PGB, and PGP on PA‐induced renal injury‐related serum enzyme markers in rats. All values represent mean ± *SEM*. Statistical analysis was performed using GraphPad Prism 5.03 (GraphPad Software, Inc) and Microsoft Excel v.2013. *p* < .05 was considered significant. *As compared to the control group. ^#^As compared to the PA group, *n* = 8

Several natural products have recently been shown to act as effective antioxidants, which are described as chemopreventive agents against free radical‐related diseases induced by numerous oxidative stresses. A large body of research has suggested that xenobiotic compounds trigger the generation of different free radicals that could give rise to several serious diseases. The levels of major biochemical markers in serum could be measured to demonstrate different parameters associated with hepatic and renal injuries. Any amelioration in the levels of hepatic and renal enzymes because of oxidative stress is considered one of the major indices of liver and renal damage (Jamakala & Rani, 2015). The major biomarkers of hepatocytic injury include elevated levels of intracellular enzymes such as ALT, AST, GGT, and ALP. Meanwhile, the biomarkers for renal tissue damage include calcium, uric acid, urea, and albumin. Increase in the levels of these enzymes and other biomarkers is indicative of cellular damage and loss of the integrity of cell membranes in liver and kidney, which might be associated with hepato‐ and renal necrosis (Charlton, Portilla, & Okusa, 2014; Nallagangula, Nagaraj, Venkataswamy, & Chandrappa, 2018). PA is a neurotoxic agent that can pass through the BBB, gut, liver, and CNS. Oral administration of PA results in dramatic changes in the biomarker levels in serum, when compared to the control conditions (Choi et al., 2018). The results of our study were in agreement with previously reported data. Pre‐oral administration and postoral administration of PA in rats induce significant elevation in various parameters of the lipid profiles such as TC, TG, and LDL‐C, and a significant reduction in HDL‐C (*p* < .05). A significant increase in the levels of liver biomarker enzymes ALT, AST, and GGT (*p* < .05) was also observed. In addition, a significant increase in renal biomarkers such as calcium, uric acid, urea, and albumin (*p* < .05) (Figures 2, 3, 4) was also observed.

Pretreatment with BP at a dose of 250 mg/kg PGE, PGB, and PGP (groups III, IV, and V) significantly reduced the PA‐induced elevated levels of serum ALT and AST (*p* < .05), whereas ALP showed no change except a significant increase in the group IV (PGB) only. Unfortunately, serum GGT was significantly increased in all fractions when compared to the groups I and II (Figure 2a). These results demonstrate the ability of different fractions of BP to restore the normal function of hepatocytes injured by PA. Furthermore, pretreatment groups III, IV, and V exhibited a remarkable decrease in the levels of TC, TG, and LDL‐C, as well as significantly increase the level of HDL‐C in the groups III and IV when compared to the PA‐treated group (*p* < .05) (Figure 3a). While post‐treatment with BP at a dose of 250 mg/kg of PGE, PGB, and PGP (groups VI, VII, and VIII) significantly reduced the PA‐induced elevated levels of serum ALT and AST (*p* < .05), ALP exhibited a significant increase in the groups VI and VIII. Unfortunately, serum GGT was significantly increased in all fractions when compared to the groups I and II (Figure 2b). However, the administration of different fractions of the PA‐treated rats (post‐treatment) showed no significant changes in all values (TC, TG, HDL‐C, and LDL‐C) when compared to the PA, while HDL‐C level in the group VII showed slight improvement (Figure 3b).

Oral feeding of rats with BP fractions (groups III, IV, V, VI, VII, and VIII) at a dose of 250 mg/kg was found to inhibit the PA‐induced high level of urea in the III, IV, VII, and VIII groups when compared to the control and PA‐treated groups (*p* < .05). However, there was no effect of all fractions on the PA‐induced elevated uric acid levels when compared to the PA. Also, the calcium levels were increased in the III, IV, V, and VIII groups when compared to the control and PA groups. Pretreatment with BP fractions (III, IV, and V) showed significant reduction in albumin levels of PGB (group IV, *p* < .05) only, while other fractions (III and V) did not induce any change when compared to the PA (Figure 4a). Moreover, post‐treatment with different fractions of BP (VI, VII, and VIII) showed significant reduction in albumin with PGE and PGP fractions (*p* < .05), while PGB fraction did not induce any change (Figure 4b).

**FIGURE 5:**
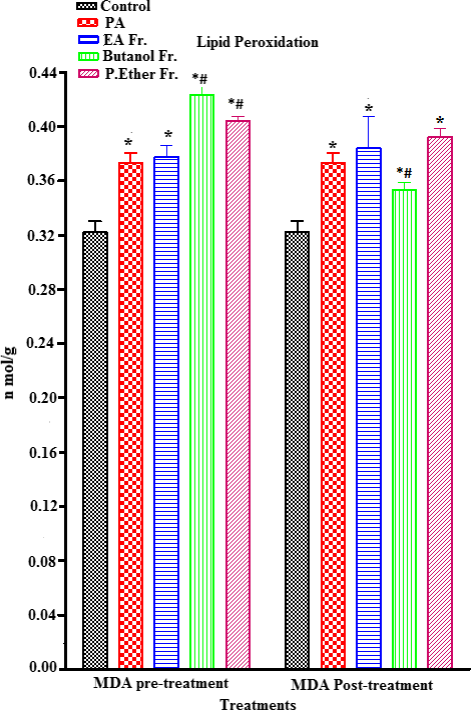
Effect of PA‐induced lipid peroxidation in rat brain homogenates. Values represent mean ± *SEM*. Statistical analysis was performed using GraphPad Prism 5.03 (GraphPad Software, Inc) and Microsoft Excel v.2013. *p* < .05 was considered significant. *As compared to the control group. Group, *n* = 8

**FIGURE 6:**
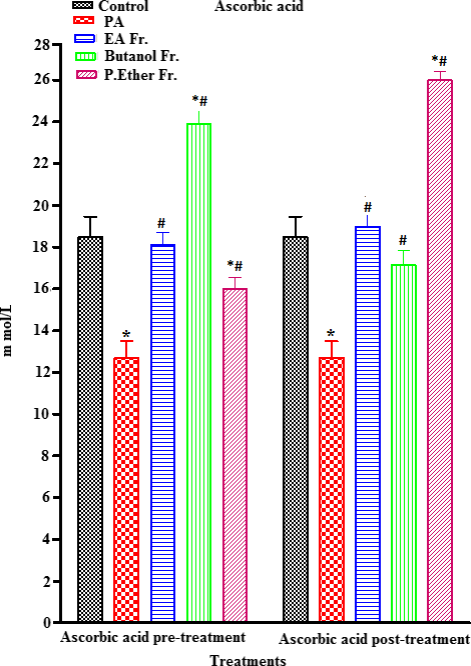
Effect of pre‐ and post‐treatment with PGE, PGB, and PGP on ascorbic acid in rats. All values represent mean ± *SEM*. Statistical analysis was performed using GraphPad Prism 5.03 (GraphPad Software, Inc) and Microsoft Excel v.2013. *p* < .05 was considered significant. *As compared to the control group. ^#^As compared to the PA group, *n* = 8

**FIGURE 7:**
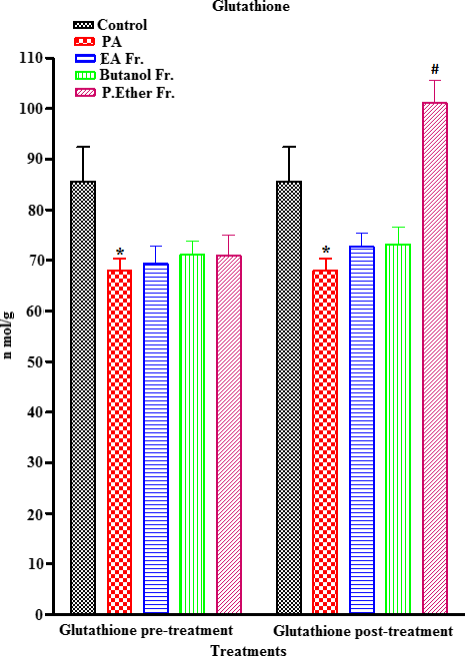
Effect of pre‐ and post‐treatment with PGE, PGB, and PGP on GSH on rats. All values represent mean ± *SEM*. Statistical analysis was performed using GraphPad Prism 5.03 (GraphPad Software, Inc) and Microsoft Excel v.2013. *p* < .05 was considered significant. *As compared to the control group. ^#^As compared to the PA group, *n* = 8

**FIGURE 8:**
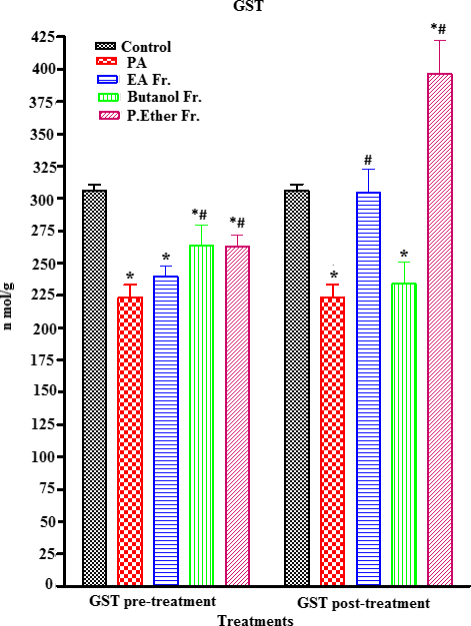
Effect of pre‐ and post‐treatment with PGE, PGB, and PGP on GST in rats. All values represent mean ± *SEM*. Statistical analysis was performed using GraphPad Prism 5.03 (GraphPad Software, Inc) and Microsoft Excel v.2013. *p* < .05 was considered significant. *As compared to the control group. ^#^As compared to the PA group, *n* = 8

**FIGURE 9:**
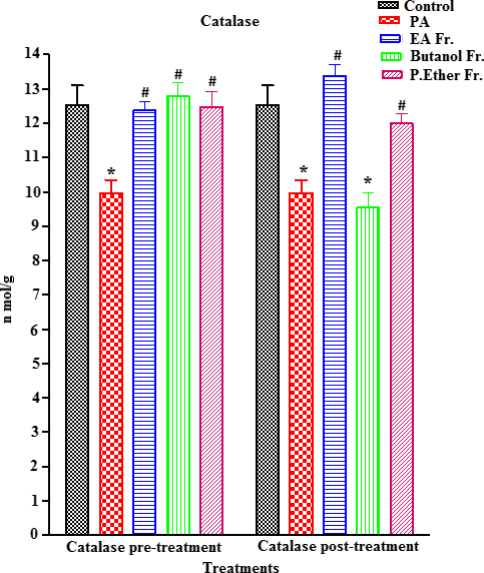
Effect of pre‐ and post‐treatment with PGE, PGB, and PGP on CAT on rats. All values represent mean ± *SEM*. Statistical analysis was performed using GraphPad Prism 5.03 (GraphPad Software, Inc) and Microsoft Excel v.2013. *p* < .05 was considered significant. *As compared to the control group. ^#^As compared to the PA group, *n* = 8

PA‐treated rats showed a significant increase in MDA (an end metabolite of lipid peroxidation) (an end metabolite of lipid peroxidation) (µmol/ml) (*p* < .05) (an end metabolite of lipid peroxidation), when compared to the control group (Figure 5). On the other hand, a concomitant significant reduction (*p* < .05) in ascorbic acid, GSH, GST, and CAT enzymes was observed, when compared to the control (Figures 6, 7, 8, 9).

In pretreatment groups, the MDA levels were significantly increased in PGB and PGP, whereas PGE was not changed in MDA level. Nevertheless, post‐treatment with the same fractions was not significantly increased in the MDA level, with a significant decrease in MDA level in a PGB fraction (Figure 5).

Prefeeding rats with BP fractions (groups III, IV, and V) at a dose of 250 mg/kg resulted in a significant increase in the PA‐induced reduction in the levels of ascorbic acid (Figure 6), GSH (no significant) (Figure 7), GST (Figure 8), and CAT (Figure 9). In addition, postfeeding of rats with BP fractions (groups VI, VII, and VIII) at a dose of 250 mg/kg resulted in a significant increase in the levels of ascorbic acid (all groups), GSH (only significant in the group VIII), GST (except group VII), and CAT (except group VII) when compared to the PA.

Furthermore, the elevation in lipid peroxide radicals may produce changes in the lipid profile and metabolism as well as induce oxidative damage to the liver, kidneys, and nucleotides. The end product of polyunsaturated fatty acid peroxidation is TBARS (thiobarbituric acid reactive substances), and MDA is a biomarker of lipid peroxidation (Catala & Diaz, 2016). We observed a significant increase in the levels of MDA, suggesting that PA causes a significant elevation in MDA (*p* < .05) in rat brain homogenates when compared to the control condition (Figure 5). Elevation in the level of lipid peroxidation in PA‐treated rats indicates brain dysfunction, which is a consequence of the production of highly reactive species.

In some conditions, the protein/carbohydrate ratio of a meal might affect neurotransmitter (NT) concentration in the brain. We therefore speculate that NTs could be affected by precursor availability or other peripheral factors that are governed by food consumption (Rui, 2013). Nonenzymatic ascorbic acid is known as a scavenger of various free radicals. We observed a reduction in ascorbic acid levels in the PA‐treated groups when compared to the control (*p* < .05). Our findings were supported by several previous studies that a reduction in ascorbic acid levels in plasma, liver, and brain homogenates could cause tissue damage (Al‐Diahan & Bhat, 2015). However, oral feeding of rats with BP upon pretreatment with PA resulted in a significant increase in the level of ascorbic acid (*p* < .05) when compared to the PA group, especially in case of the PGB fraction that showed a significant increase in ascorbic acid levels when compared to the control (*p* < .05). On the other hand, post‐treatment with PGP fraction exhibited a higher level of ascorbic acid in all treated groups when compared to the control and PA groups (*p* < .05; Figure 6).

Furthermore, the levels of nonprotein thiols (NP‐SH) such as GSH, nonenzymatic antioxidants, and antioxidant enzymes (GST and CAT) exhibit a reduction in tissues, due to their rapid consumption upon exposure to oxygen reactive species. Therefore, preventing the generation of intracellular free radicals could play a pivotal role in protecting against liver, kidney, and brain diseases. Antioxidants have the ability to inhibit oxidative stress and free radical formation in our bodies (Kurutas, 2016). GSH possesses a pivotal role in maintaining the normal structure and function of cells, and is considered as the most powerful reductant and detoxifying agent in the cell that protects against peroxides and other xenobiotics (Shelly & Lu, 2013). We detected a significant reduction in GSH levels (*p* < .05) in brain homogenates in PA‐treated rats when compared to the control. This could be due to the oxidation of GSH to GSSG and the reduction in NADPH because of the antioxidant effects. Our results were in agreement with the previously reported studies showing that an increase in the level of PA caused oxidative stress by lowering GSH levels in brain tissues (Rose et al., 2012). However, pre‐ and post‐treatment with BP showed a slight, nonsignificant recovery of all treatment groups (III, IV, V, VI, and VII) expect the PGP fraction (group VIII) that showed a highly significant increment (*p* < .05) when compared to the control (group I) (Figure 7). Moreover, GST is considered a group of isoenzymes that detoxifies xenobiotic free radicals by conjugating with GSH. Any decrease in GST activity triggers an overload of highly reactive oxygen species that leads to injury to many vital cells (Qu, Chen, Hu, & Feng, 2016). In our study, a significant reduction in GST (*p* < .05) was observed in the case of PA‐treated rats when compared to the control group (Figure 8). The observed reduction in GST levels upon exposure to PA is in agreement with previous work that supports our finding (Alfawaz, Bhat, & Al‐Ayadhi, 2014). Orally fed rats with BP pretreatment (groups III, IV, and V) with PA showed a significant increase in the level of GST (*p* < .05) when compared to the PA. However, PGE and PGP fractions (groups VI and VIII) exhibited a higher level of GST in the post‐treatment group when compared to the control (*p* < .05) (Figure 8).

In addition, catalase (CAT) is a potent enzyme that scavenges the reactive oxygen species produced due to oxidative stress (Cruz de Carvalho, 2008). The results of our study show significant reduction in CAT levels (*p* < .05) in brain homogenates of PA‐treated rats when compared to the control (Figure 9). Nevertheless, a significant increase in CAT levels that was detected in all treatment groups (except group VII) when compared to the PA, especially in case of the PGE post‐treatment group (VI), illustrates a potent activity against the PA‐induced reduction in CAT enzyme (*p* < .05).

Reduced levels of antioxidants such as GSH, GST, and CAT were reported in rat testis after oral administration of tuberculosis (TB) drugs. In their study, a significant increase in GSH, GST, and CAT activities was reported after BP administration along with TB drugs (Bharti, Kumar, & Kaur, 2017). Another study conducted by Shayakhmetova, Bondarenko, and Kovalenko (2012) reported that rats administered with TB drugs showed increased levels of epithelium exfoliation into seminiferous tubules (Shayakhmetova et al., 2012). All these studies provide evidence for the presence of multiple antioxidant components in BP, which is considered a promising source of new antimicrobial agents. In addition, BP is reported to have protective effects against carbon tetrachloride and malathion‐induced toxicities (Kandiel, El‐Asely, Radwan, & Abbass, 2014; Yildiz et al., 2013). Collectively, our results clearly point out toward the efficacy of BP in conferring protection against PA‐induced toxicity and show that supplementation of animal diet with BP exhibits a protective effect against various antibiotics. We therefore believe that BP could be considered a potential protective agent against drug‐induced toxicity in rats, as exhibited by its ability to rebuild the redox status within tissues. The overall hepatorenal and brain protection might be due to BP fractions, which contain high amount of polyphenols, flavonoids, and cholinergic acids, which have been reported to possess high antioxidant potential owing to their ability to attack reactive oxygen species (Ahmed et al., 2015; Lobo, Patil, Phatak, & Chandra, 2010). This could lead to in vivo inhibition of lipid peroxidation along with the restoration of protein expression and NP‐SH system. Moreover, the high flavonoid content in different fractions could effectively suppress PA‐induced toxicity.

Based on these results, BP could be recommended as an aliment strategy for autistic children, especially for those complaining of detoxification deficiencies, suffering from chronic inflammation and abnormal gut microbiota (Al‐Abdali, Al‐Ayadhi, & El‐Ansary, 2014). This suggestion is supported by some recent studies, which revealed the capability of natural BP in the recovery of autism induced by *Clostridium perfringens* in vitro, and the detoxifying anti‐inflammatory, and antimicrobial effects of BP (Al‐Yousef, Alkhulaifi, Al‐Salem, & Syed, 2018; Choi, 2007; Kroyer & Hegedus, 2001). These effects could be a consequence of the presence of different chains of fatty acids, and phenolic and proanthocyanidin components present in abundance in different fractions of BP (Aabed, Shafi Bhat, et al., 2019; Al‐Yousef et al., 2018).

## 4 CONCLUSION

Phyto‐ and biochemical results of pollen bee fractions exhibited remarkable protective effects of BP against the PA‐induced hepatorenal and brain injuries. These protective effects may be due to the presence of phenolic compounds, proanthocyanidins, flavonoid, and its glycosides in the BP fraction. These can exhibit strong free radical scavenging and antioxidant activities that suppress oxidative stress induced by different xenobiotics including PA. This effect can also play an important role in maintaining normal physiological features of the organs. Our findings demonstrate that BP may serve as a prophylactic and/or an adjuvant therapy in the treatment of various chronic insufficiencies induced by oxidative stress related to liver, kidney, and brain involved in the etiology of autistic features. This is suggestive of the possible therapeutic relevance of BP. We believe that its apparent safety over long‐term administration is encouraging enough to warrant further studies to explore its possible role in modern clinical practice.

## CONFLICT OF INTEREST

The authors declare that they do not have any conflict of interest.

## ETHICAL APPROVAL AND CONSENT TO PARTICIPATE

The protocols used in this study were approved by the Ethics Committee of Experimental Animal Care Society, King Saud University, and all the experiments were performed in accordance with the guidelines of the National Animal Care and Use Committee, Saudi Arabia.
